# Production and secretion of recombinant spider silk in *Bacillus megaterium*

**DOI:** 10.1186/s12934-024-02304-5

**Published:** 2024-01-26

**Authors:** Alexander Connor, R. Helen Zha, Mattheos Koffas

**Affiliations:** 1 Department of Chemical and Biological Engineering, Rensselaer Polytechnic Institute, Troy, NY 12180 USA; 2 Center for Biotechnology and Interdisciplinary Studies, Rensselaer Polytechnic Institute, Troy, NY 12180 USA

## Abstract

**Background:**

Silk proteins have emerged as versatile biomaterials with unique chemical and physical properties, making them appealing for various applications. Among them, spider silk, known for its exceptional mechanical strength, has attracted considerable attention. Recombinant production of spider silk represents the most promising route towards its scaled production; however, challenges persist within the upstream optimization of host organisms, including toxicity and low yields. The high cost of downstream cell lysis and protein purification is an additional barrier preventing the widespread production and use of spider silk proteins. Gram-positive bacteria represent an attractive, but underexplored, microbial chassis that may enable a reduction in the cost and difficulty of recombinant silk production through attributes that include, superior secretory capabilities, frequent GRAS status, and previously established use in industry.

**Results:**

In this study, we explore the potential of gram-positive hosts by engineering the first production and secretion of recombinant spider silk in the *Bacillus* genus. Using an industrially relevant *B. megaterium* host, it was found that the Sec secretion pathway enables secretory production of silk, however, the choice of signal sequence plays a vital role in successful secretion. Attempts at increasing secreted titers revealed that multiple translation initiation sites in tandem do not significantly impact silk production levels, contrary to previous findings for other gram-positive hosts and recombinant proteins. Notwithstanding, targeted amino acid supplementation in minimal media was found to increase production by 135% relative to both rich media and unaltered minimal media, yielding secretory titers of approximately 100 mg/L in flask cultures.

**Conclusion:**

It is hypothesized that the supplementation strategy addressed metabolic bottlenecks, specifically depletion of ATP and NADPH within the central metabolism, that were previously observed for an *E. coli* host producing the same recombinant silk construct. Furthermore, this study supports the hypothesis that secretion mitigates the toxicity of the produced silk protein on the host organism and enhances host performance in glucose-based minimal media. While promising, future research is warranted to understand metabolic changes more precisely in the *Bacillus* host system in response to silk production, optimize signal sequences and promoter strengths, investigate the mechanisms behind the effect of tandem translation initiation sites, and evaluate the performance of this system within a bioreactor.

**Supplementary Information:**

The online version contains supplementary material available at 10.1186/s12934-024-02304-5.

## Introduction

Silk proteins are a class of protein-based biomaterials that have garnered significant interest due to their unique combination of chemical and physical properties, varied morphologies, and diverse applications [1–3]. Two of the most extensively studied categories of silk protein include the cocoon silk of domesticated *Bombyx mori* silkworms and dragline silk from orb-weaving spiders. Dragline silk is used by orb-weaving spiders to form the sturdy framework of their webs, and it is renowned for its robust mechanical properties. Indeed, dragline spider silk from *A. diadematus* is only 1/6 the density of steel but exhibits 30 times the toughness (energy to break) [3]. Additional work has shown that the valuable attributes of spider silk proteins (spidroins) extend far beyond mechanical properties. In general, silk proteins are biodegradable, thermally stable, lightweight, and display a high level of biocompatibility with humans [3–6]. Importantly, silk proteins can be processed into a diverse array of morphologies that includes hydrogels, coatings, films, tissue scaffolds, adhesives, and nano-patterned photonic materials [7–9]. Research has also shown that the properties of materials fabricated from natural or recombinant silks are tunable, with alterations to the primary sequence (in the context of recombinant silk) and processing conditions of the proteins modulating parameters such as strength, extensibility, and macromolecular conformation in a targeted way [3, 4, 10–14]. As such, the potential applications of silk range from surgical sutures to optofluidic devices and even coatings for food preservation [6, 9, 15]. Therefore, studies examining the production of silk proteins represent an imperative aspect of research necessary to capture the full potential of protein-based biomaterials.

In contrast to silk proteins produced by the domesticated *Bombyx mori* silkworm, spider silk cannot be farmed from spiders at scale [16]. To date, recombinant production of spider silk proteins has been identified as the most promising route to establish industrial scale production [17, 18]. Recombinant spider silk has been produced with varying degrees of success in a diverse set of host organisms that includes bacteria, yeast, mammalian cells, insect cells, transgenic plants, and transgenic animals [19, 20]. Most efforts to produce recombinant spidroins have suffered from suboptimal titers, largely preventing the production and utilization of spider silk at a commercial scale [17, 21]. Additionally, expressing recombinant spidroins in both prokaryotic and eukaryotic hosts is often plagued by a high degree of plasmid instability, toxicity to the host, low solubility of the spidroin constructs, and transcriptional and translational errors [20, 22–26]. Notwithstanding, two recent works have shown traction regarding high titers of recombinant spidroins. Using an *E. coli* host system, Yang et al. achieved a titer of 3.6 g/L for 200 kDa dragline spidroin in a bioreactor kept at 16 ℃. The researchers also employed a secondary plasmid to upregulate glycyl-tRNA supply [22]. Likewise, Schmuck et al. documented a titer of 14.5 g/L for a miniature recombinant spidroin construct (NT2RepCT) using an *E. coli* host system in a bioreactor [27]. However, increasing titer alone will likely not lower the cost of production enough for recombinant silk to be an economically viable material outside of markets that can support high price points (ex. biomedical applications) [21]. The harvesting and lysis of *E. coli* cells, in combination with the purification methods used in the aforementioned reports (e.g. affinity chromatography), is theorized to constitute approximately 50% of the operating cost of a scaled production plant, independent of the spidroin titer [21]. In addition, the use of an *E. coli* host can pose issues for applications of silk proteins in which a Generally Regarded as Safe (GRAS) host would be preferred if not required, such as the biomedical and food industries.

Within this scope, gram-positive bacteria are an attractive option to produce recombinant silk due to their GRAS status, widespread use in industry, and strong secretion capabilities [28]. Secretion of recombinant silk is of particular interest, as it may greatly reduce cell harvesting, lysis, and purification costs at scale. Moreover, recent work has demonstrated that intracellularly produced recombinant silk proteins can exert toxicity on a host organism, yielding suboptimal biomass growth and low titers in cost-effective minimal media [26, 29]. Despite these potential benefits, gram-positive hosts and bacterial secretion are underexplored within the recombinant silk field. To our knowledge, a 2022 report using a *C. glutamicum* host is the only study that employs silk production and secretion in a gram-positive species [30]. Promising results, that included a secreted titer in bioreactors of 554 mg/L with a 93% purity (without chromatography), indicate that research efforts should be allocated in this direction.

Here, we report the first production and secretion of recombinant silk in the *Bacillus* genus by way of an industrially relevant *B. megaterium* host. Findings include that the previously observed toxicity that intracellularly produced recombinant silk exerts on a host organism appears to be mitigated when secretion is employed [26]. Furthermore, data shows that metabolic bottlenecks observed in *E. coli* during recombinant silk production may extend across the phylogenic tree and exist in *B. megaterium*. Targeted media supplementation that addresses such metabolic bottlenecks was found to yield secreted recombinant silk titers of 98.2 ± 6.5 mg/L, representing the highest reported secreted silk titers for a bacterial host in shake flasks to our knowledge. These shake flask production levels are also approximately equal to the aforementioned and efficiently scaled NT2RepCT protein, and future work will seek to analyze the performance of this new *B. megaterium* host system in a bioreactor. [27]. Additionally, an investigation of altered (in tandem) translation initiation sites (TISs) was found to offer no benefit for silk production levels, contrasting previous work that demonstrated the utility of tandem TISs across multiple gram-positive hosts and recombinant proteins [31]. Thus, this work establishes a novel secretory production platform for recombinant silk and offers a preliminary investigation of its potential as an industrial platform.

## Results and discussion

### Secretory production of spider silk in *B. megaterium*

All strains used in this work were constructed from a *B. megaterium* MS941 parent strain. *B. megaterium* MS941 is a derivative of the DSM319 strain in which the only major extracellular protease has been inactivated [32]. *B. megaterium* is an attractive host choice for this work as it contains no endotoxins and can utilize inexpensive feedstocks [33, 34]. The species can also maintain recombinant plasmids well, with xylose-inducible promoters previously used to achieve high levels of recombinant protein secretion [35–39]. Moreover, *B. megaterium* has been an industrial work horse for over 50 years, producing a range of compounds that includes vitamin B12, penicillin precursors, enzymes (ex. glucose dehydrogenase and amylase), pyruvate, and fungicidal molecules [33, 40]. A list of all strains, plasmids, signal sequences, translation initiation sequences, and the recombinant silk sequence used in this work can be found in Additional file 1: Table S1.

We have previously produced the A5 4mer recombinant silk protein in *E. coli*, and this construct was selected for secretory silk production in *B. megaterium* [26]. Naturally occurring dragline spider silk fibers contain two spidroin proteins, major ampullate 1 (MaSp1) and major ampullate 2 (MaSp2) [41]. The A5 4mer is a small, 16 kDa protein with a synthetically designed primary sequence that mimics the natural structure of MaSp2 [26]. To prevent excess amino acids in the recombinant silk construct from interfering with secretory mechanisms, the following changes were made in the primary sequence of the version produced in *B. megaterium* versus that of *E. coli* (Additional file 1: Table S1) [42]. The construct produced in *B. megaterium* lacks the 18-residue spacer (which is not a spidroin-based sequence) between the histidine tag and the start of the A5 4mer primary sequence. Additionally, the final four amino acids found in the *E. coli* A5 4mer version were spacer residues (not part of the designed silk sequence) present on the plasmid backbone that used in that work and have been removed from the version produced in *B. megaterium*. Furthermore, the A5 4mer produced in *B. megaterium* has a histidine tag that has been shortened from 10 tandem histidine residues to 6 (Additional file 1: Table S1). Moreover, the histidine tag on the construct produced in *B. megaterium* was moved from the N-terminus to the C-terminus to help facilitate Sec secretion. This new A5 4mer gene was cloned into a panel of five different *B. megaterium* expression vectors that each contained a unique secretion signal sequence (SS) and five amino-acid longer linker prior to the start of the A5 4mer gene (Additional file 1: Table S1). These SSs, namely α-amy, LipA, NprM, Yoch, and Yngk, were previously found to facilitate the production and secretion of recombinant cellulases in *B. megaterium* and were chosen for the initial screening of recombinant silk secretion (Additional file 1: Table S1) [35]. Each SS directs for the recombinant silk to be secreted through the Sec secretion pathway [35]. Due to the presence of only one cell membrane layer, secretion of recombinant proteins into the culture media by a gram-positive species can be achieved with the Sec secretion pathway. In contrast, gram-negative hosts must utilize the more complex TaT or Type 1–6 pathways to secrete products into the culture media [35, 36, 42]. Therefore, utilization of the Sec pathway in gram-positive hosts is biologically simpler, theoretically more efficient for recombinant proteins, and requires only the addition of a short N-terminal SS on the protein of interest rather than more extensive engineering of genetic circuits [35, 36, 42]. Indeed, secretory production of recombinant silk in the gram-negative *S. typhimurium* using a type III secretion pathway was found to have severe shortcomings. Achieving secretion required the implementation of a multifaceted genetic circuit that modulated chaperone proteins, cellular secretion needles, and protease activity. Ultimately, only 14% of expressed protein was secreted and the secreted titer was only 1.8 mg/L*h [43].

**Fig. 1:**
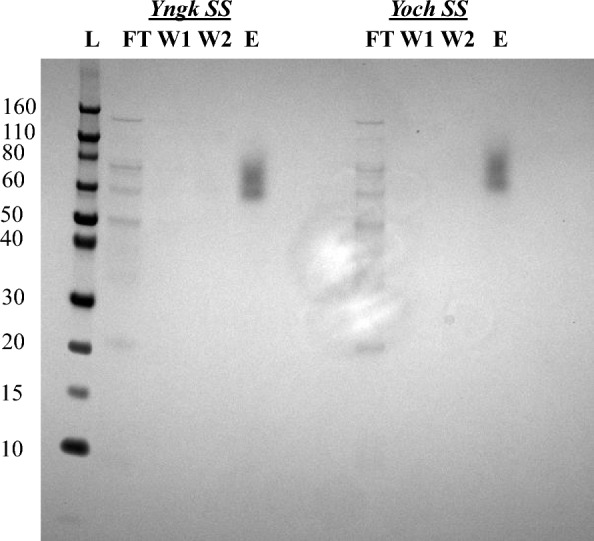
SDS PAGE from nickel-chromatography purification on supernatants of strain Yngk and Yoch. Lanes: (L) Protein ladder with kDa values listed to the left. (*Yngk SS)* Flow through of Yngk (FT), Wash 1 of Yngk (W1), Wash 2 of Yngk (W2), Elution of Yngk (E). (*Yoch SS)* Flow through of Yoch (FT), Wash 1 of Yoch (W1), Wash 2 of Yoch (W2), Elution of Yoch (E). The purified A5 4mer silk can be found in the elution lanes for both SSs (at approximately 60 kDa). Inefficient Coomassie staining of the secreted A5 4mer protein was observed

Preliminary experiments were performed in LB media at 37 °C in shake flasks. The supernatants from expressions cultures were put through nickel-chromatography and SDS PAGEs were performed to identify the presence of secreted A5 4mer silk protein. As shown in Fig. 1, the Yngk and Yoch SSs enabled successful secretion of the silk protein (strains Yngk and Yoch). However, the α-amy, LipA, and NprM SSs did not yield any detectable secreted product. Despite up to fivefold concentrations of the elution fractions for these SSs, secreted protein was not detected by SDS PAGE, therefore, these strains were not included in Fig. 1. For all SSs tested, no concurrent intracellular production was observed, with all silk protein produced in the host secreted.

It was observed through SDS PAGE that the version of the A5 4mer produced in *B. megaterium* exhibited different behavior versus that of *E. coli.* The version produced in previous studies using *E. coli* showed an apparent molecular weight on SDS PAGE of 38 kDa (actual molecular weight of 16 kDa) due to its high level of intrinsic disorder [26, 44, 45], while the *Bacillus* version can be seen at approximately 60 kDa (identical SDS PAGE procedures were used) (Fig. 1). This increase in apparent molecular weight is not due to the presence of a SS, as that is cleaved from a protein during Sec secretion [42]. Notwithstanding, future work could implement LC/MS–MS techniques to confirm that assumption for this system. Rather this difference in apparent molecular weight (increase in aberrant SDS PAGE mobility) may indicate an increased level of intrinsic disorder in the *Bacillus* construct [26, 44, 45]. Moreover, the *Bacillus* A5 4mer shows weak staining with Coomassie blue (Fig. 1). In addition, computational analysis of the A5 4mer in previous work showed that for all residues in the sequence there is near 100% probability that they are located in regions of structural disorder [26]. These differences are likely due to the slight changes in primary sequence between the *Bacillus* and the *E. coli* A5 4mer constructs. Specifically, truncation of a histidine tag has been found to increase structural disorder within proteins (Additional file 1: Table S1) [46]. Likewise, Coomassie blue interacts primarily with arginine, tyrosine, lysine, and histidine residues, and the histidine tag on the A5 4mer sequence is the only portion of the peptide that contains any of these amino acids [47].

**Fig. 2:**
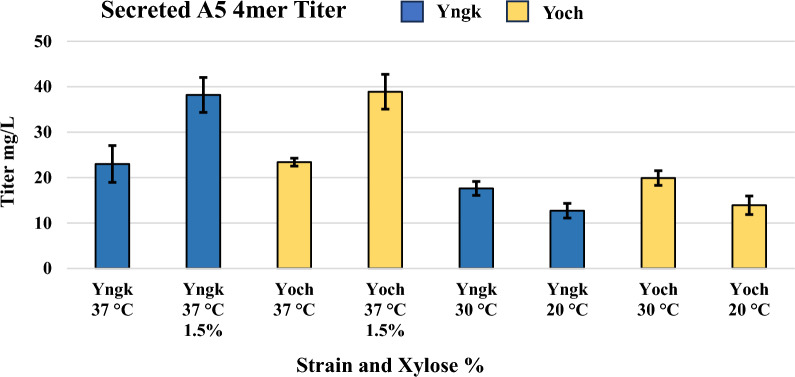
Secreted A5 4mer titer in Yngk and Yoch strains at various expression temperatures and xylose (inducer) concentrations in LB media. Induction occurred when cells reach an OD600 of 0.275–0.325 with 0.5% w/v xylose unless otherwise noted (1.5% w/v). All expressions were carried out for 21 h. Error bars represent standard deviations from the mean values of three replicates

The Yngk and Yoch strains were tested for their secretion levels at various temperatures and xylose (inducer) concentrations in LB media (Fig. 2). The secreted titer for both strains was found to be approximately 23 mg/L at 37 °C with a 0.5% w/v xylose induction. Lowering the expression temperature to 30 °C in conjunction with a 0.5% w/v xylose induction was found to lower titers to approximately 17 mg/L and 20 mg/L for Yngk and Yoch, respectively. Likewise, lowering the expression temperature to 20 °C in conjunction with a 0.5% w/v xylose induction was found to further lower titers to approximately 12 mg/L and 13 mg/L for Yngk and Yoch, respectively. Due to this decrease in titer, only 37 °C was used for subsequent experiments. If expressions were performed at 37 °C with a stronger xylose induction (1.5% w/v) titers were observed to increase to approximately 38 mg/L for both strains, representing a 65% increase versus 0.5% w/v xylose. The increase in secreted titers with higher inducer (xylose) levels may be because this *B. megaterium* strain is able to metabolize xylose, and its depletion may lead to suboptimal titers [48]. A strain of *B. megaterium* that is unable to metabolize xylose has been previously engineered (YYBm1), however, it is incompatible with the expression vectors used in this work [49]. Future work should engineer a greater diversity of expression vectors and additional *B. megaterium* mutants unable to metabolize xylose to understand the effect of inducer concentration and consumption more clearly. However, work with strain YYBm1 has not always led to increased titers versus MS941 (strain in this work), with results dependent on additional factors outside of inducer consumption [48].

Notably, the secreted titers for strains Yngk and Yoch remain practically identical across the conditions tested thus far. This is unexpected as previous work shows that variation in the SS can result in substantial changes in the secreted titer of a recombinant protein, including order of magnitude differences [34, 35]. The A5 4mer is a small (~ 16 kDa) and disordered recombinant protein, and the Sec secretion machinery is particularly efficient at secreting small proteins in an unfolded state [42]. This, combined with the observation that none of the strains used in this work exhibit concurrent intracellular silk production indicates that secretion may not be a bottleneck for small, disordered spidroin production in *B. megaterium*. Alternatively, other bottlenecks such as metabolic burden or translational difficulties may be involved.

To work towards an improved understanding of this host system and to compare intracellular to secreted production, an additional expression strain was created in which the Yoch SS and linker sequence were removed from the expression vector. Therefore, this strain should produce an identical version of the A5 4mer silk protein but retain the construct intracellularly. However, production of the silk construct was not detected by SDS PAGE or by titer quantification methods for this non-secreting strain. As such, it was hypothesized that this strain did not produce any silk protein during experiments. This finding inhibited the ability to compare intracellular and secreted production for *B. megaterium* but is significant in showing the potential necessity of secretion to produce structurally disordered silk constructs in certain species.

### Investigation of altered translation initiation sites (TISs)

The Yoch strain in conjunction with expressions in LB media at 37 °C with 1.5% w/v xylose was chosen as a starting point for subsequent work that aimed to increase secreted silk titer and identify potential bottlenecks within the system. Emerging work within synthetic biology has identified translation efficiency as a common rate-limiting step for protein production [50]. Translation efficiency depends on multiple factors, including the speed of translation elongation and the rate at which translation is initiated [31, 50]. Translation initiation specifically refers to the binding of a ribosome to mRNA at the ribosome binding site (RBS) followed by the scanning of the ribosome along the mRNA’s untranslated region to identify a start codon and initiate translation [51]. The RBS, untranslated region, and start codon can together be referred to as the translation initiation site or TIS [52]. This is the first step in the translation process and is of particular interest for recombinant protein production in gram-positive bacteria. Previous work has found that multiple TISs on a gene can all be active during translation in gram-positive bacteria, however, only one TIS can be active for a given gene in the gram-negative *E. coli* [52]. It is hypothesized that in gram-positive bacteria multiple tandem TISs can engage multiple ribosomes on a single mRNA strand during translation, potentially increasing the efficiency of recombinant protein production [31]. Indeed, recent work has shown that designing recombinant genes with multiple tandem TISs leads to higher recombinant protein titers across multiple gram-positive species. The effect was found to be correlated to the number of TISs, with increased levels of GFP production in *B. subtilis* and *C. glutamicum* as the number of TISs increased over a range of 1–6 [31]. However, multiple TISs were not favorable for increased GFP production in *E. coli* and could even lower titers in some cases [31]. Other work also supports the notion that translation initiation is a powerful lever in gram-positive bacteria. Xiao et. al designed a set of modular TISs that were predicted to form various hairpin structures upon transcription of the gene to mRNA [53]. These hairpin structures were theorized to increase the stability of the mRNA by protecting it from exonucleases while simultaneously promoting translation initiation by improving the accessibility of the ribosome binding site and start codon. The mRNA structures were designed to expose the RBS on the loop of the hairpin structure and contain a start codon on, or directly after, the stem [53]. These engineered TISs improved GFP and nattokinase production in *B. licheniformis* by 50-fold and 13-fold, respectively [53]. Interestingly, application of these engineered TISs in gram-negative *E. coli* produced negligible or negative changes for GFP production [53].

These previous findings served as the basis for subsequent experiments that used variations of the Yoch strain. Five new strains were designed to understand if multiple tandem TISs or TISs designed to form a hairpin structures could increase titers of secreted silk (Additional file 1: Table S1). Due to a lack of significant differences in titer or growth between the Yngk and Yoch strains across multiple temperatures and xylose levels, the Yoch SS was randomly chosen for the following experimental design. Two of the strains, OG3x and OG6x, contained the original translation initiation nucleotide sequence used in this work (inclusive of the ribosome binding site and start codon) that had been extended in tandem to include three or six discrete copies of the entire translation initiation sequence, respectively. A different strain was created (UTR1x) in which a single copy of a new translation initiation site was swapped for the original. This new translation initiation site (UTR) is a slightly altered version of a TIS sequence previously designed to form a hairpin structure [53]. Two final strains were created, UTR3x and UTR6x, in which the translation initiation site present on UTR1x was extended in tandem (inclusive of the ribosome binding site and start codon) to include three or six discrete copies of the entire translation initiation sequence, respectively. The five new strains (OG3x, OG6x, UTR1x, UTR3x, and UTR6x) all contained a single Yoch SS and Yoch linker sequence after the translation initiation site(s), followed by a single copy of the A5 4mer recombinant silk gene, forming a genetic configuration that mimics previous work [31].

**Fig. 3:**
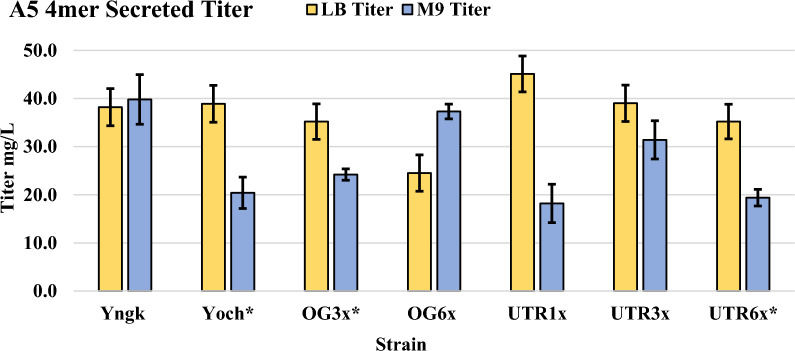
A5 4mer secreted titer for strains with altered TISs compared to strains Yngk and Yoch in LB and M9 media. The M9 minimal media was supplemented with 1% w/v glucose as a carbon and energy source. Strains with an asterisk (*) were unable to grow overnight in M9 media when cultures were inoculated from a single colony, these strains were also observed to have an extended lag phase when expression cultures in M9 minimal media were inoculated from overnight cultures grown in LB media. All data was generated from 21-h expressions at 37 °C with 1.5% w/v xylose supplemented for recombinant gene induction. Error bars represent standard deviations from the mean values of three replicates

Expressions were carried for 21 h at 37 °C in LB media with 1.5% w/v xylose supplemented for recombinant gene induction. Results show that secreted titers with the altered translation initiation sites varied from a low of 24.5 ± 3.8 mg/L with the OG6x strain to a high of 45.1 ± 3.7 mg/L with the UTR1x strain (Fig. 3). The titer of 45.1 ± 3.7 mg/L for strain UTR1x is approximately 15% higher than that achieved with the Yoch parent strain. However, it should be noted that this corresponds to an absolute increase in titer of only 6 mg/L and was determined to be statistically insignificant (P > 0.05). In contrast to other work, increasing the number of translation initiation sites was found to slightly decrease secreted titer in a monotonic way for both the OG and UTR sequences (Fig. 3) [31]. To our knowledge, this was the first experimentation with multiple translation initiation sites in *B. megaterium* for the production of silk constructs. Our results indicate that translation initiation is not a primary bottleneck in this system. It is possible that the unique primary sequence and structure of the A5 4mer silk protein, which differs substantially from the fluorescent proteins and enzymes used in the previous works employing TIS alterations, is a factor behind these results [31, 53]. Notable observations include that none of the five strains with altered TISs produced any intracellular silk, as all production was found in the secreted fraction. Furthermore, SDS PAGE showed that purified silk from a TIS-altered strain was found to appear at same apparent molecular weight (~ 60 kDa) as the construct produced in the parent Yoch strain (Fig. 1 and Additional file 1: Table S1). This is significant in that strains OG3x, OG6x, UTR3x, and UTR6x contain multiple in-frame start codons upstream of the Yoch SS (Additional file 1: Table S1), meaning that it is possible to have an N-terminal amino acid sequence prior to the Yoch SS on the newly translated (but not secreted) A5 4mer preprotein. Thus, it appears that the added N-terminal sequences did not affect ability of the host to secrete the construct or cleave the SS from the silk protein during Sec secretion, a finding that is in line with previous work using TISs containing multiple start codons [31]. However, it is possible that only the final start codon was active in the OG3x, OG6x, UTR3x, and UT6x strains, thus generating the same A5 4mer preprotein as the Yoch parent strain. To determine the relative activity of multiple start codons, previous work has removed the SS from vectors with tandem TISs to visualize bands on SDS PAGE of increasing weight that are dependent on the start codon used to initiate translation [31]. However, the inability of the *B. megaterium* host to produce A5 4mer silk protein in the absence of a SS prevented such analysis in this work.

### Media supplementation to increase silk production in *B. megaterium*

To further characterize this novel silk expression platform and identify how fundamental bioprocessing parameters impact outcomes a series of experiments using M9 minimal media were performed. The use of minimal media has several advantages, including that it can be considerably more cost effective at the industrial scale [21]. Additionally, its chemical composition is more precisely defined versus complex media derived from peptones, enabling more reproducible outcomes, and enhanced analytical analysis [54]. Moreover, and of particular interest, secretory protein titers in *B. megaterium* have been found to change substantially when transitioning from rich to minimal media, with both increases and decreases observed [35].

Minimal media expressions were carried out in M9 minimal media with 1% w/v glucose for strains Yngk, Yoch, OG3x, OG6x, UTR1x, UTR3x, and UTR6x, as it was of interest to see how each unique strain would respond to minimal media. All expressions were carried out for 21-h at 37 °C with 1.5% w/v xylose supplemented for recombinant gene induction. Titers in M9 media for the Yoch, OG3x, UTR1x, and UTR6x strains were approximately 20 mg/L, representing a decrease of 43–55% versus LB media (Fig. 3). The titers for strains Yngk and UTR3x in M9 remained approximately equal to those in LB, with the secreted titer of 39.8 ± 5.2 mg/L in Yngk representing the highest titer in M9 for all strains. The OG6x strain was the only strain to show a non-negligible increase in titer when transitioning from LB to M9, going from 24 ± 3.8 to 37 ± 1.5 mg/L. No concurrent intracellular production was observed for any strains in M9 media. Similar to findings in LB media, expressions in M9 continued to question the applicability of multiple TISs for *B. megaterium* and recombinant silk production. While titers increased in M9 media by 54% (+ 13 mg/L) from the OG3x to the OG6x strain, no such trend was observed for the UTR1x, UTR3x, and UTR6x strains, indicating that a correlation between the number of TISs and silk titer is not apparent. Moreover, strain Yngk (having a single TIS) yielded the highest titer out of all strains in M9 media.

**Fig. 4:**
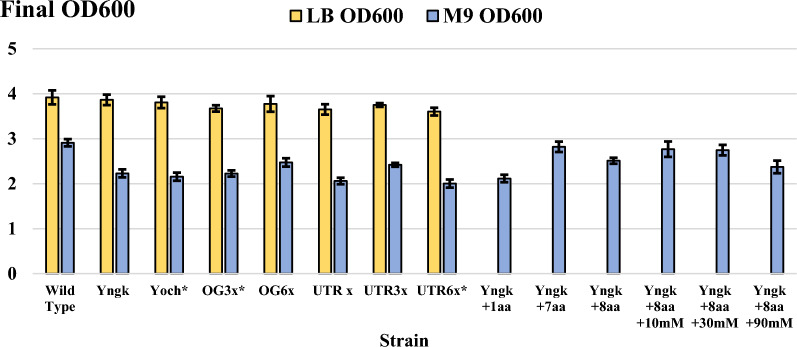
Final OD600 of strains after a 21-h expression at 37 °C with 1.5% w/v xylose supplemented for recombinant gene induction in LB and M9 media. The M9 minimal media was supplemented with 1% w/v glucose as a carbon and energy source. Strains with an asterisk (*) were unable to grow overnight in M9 media when cultures were inoculated from a single colony, these strains were also observed to have an extended lag when expression cultures in M9 minimal media were inoculated from overnight cultures grown in LB media. Additional expressions were performed with strain Yngk in altered M9 minimal media that contained 0.3 g/L of glutamate (+ 1aa), 0.3 g/L of histidine, isoleucine, phenylalanine, proline, tyrosine, lysine, and methionine (+ 7aa), 0.3 g/L of glutamate, histidine, isoleucine, phenylalanine, proline, tyrosine, lysine, and methionine (+ 8aa), 10 mM sodium acetate at time of induction (+ 10 mM), 30 mM sodium acetate at time of induction (+ 30 mM), and 90 mM sodium acetate at time of induction (+ 90 mM). Wild type corresponds to the *B. megaterium* MS941 strain lacking a silk expression vector. Error bars represent standard deviations from the mean values of three replicates

It should be noted that the strains producing titers in M9 that are equal to or exceeding those measured in LB (Yngk, OG6x, and UTR3x, shown in Fig. 3) did so while accumulating substantially less biomass in M9 (Fig. 4). The OD600 at the end of expressions in LB or M9 was consistent across all strains and was only dependent on the media used. The final OD600 in LB ranged from 3.6 to 3.86, and from 2 to 2.47 in M9 media. Importantly, the final OD600 for strains expressing and secreting the silk construct was approximately identical to the wild-type strain lacking a silk expression vector (*B. megaterium* MS941). This contrasts with our previous work, in which intracellular production of the A5 4mer in *E. coli* resulted in host toxicity, as shown by substantial decreases in growth upon induction [26]. These findings indicate that toxicity of a recombinant silk construct can be mitigated through secretion, potentially posing implications for scaled production in which obtaining high biomass levels is critical (Fig. 4) [21]. However, it was observed that certain silk expression strains exhibited poor growth when M9 overnight cultures were inoculated with a single plated colony, namely Yoch, OG3x, and UTR6x. Therefore, expression cultures in M9 media for these strains were inoculated from LB overnight cultures, however, an extended lag phase was observed in these expression cultures. Expression cultures in M9 media for the other strains reached the target OD600 for induction (0.275–0.325) within 3 h after inoculation. However, the Yoch, OG3x, and UTR6x expression cultures in M9 required approximately 6 h to reach the target OD600 for induction, despite being inoculated from dense LB overnight cultures.

Interestingly, these differences in growth after inoculation among the strains did not yield sizable differences in the OD600 at the end of expressions in M9 media (Fig. 4). Comparing the lagged growth of Yoch to the quick growth of Yngk in M9 media, it can be concluded that the SS and linker affect performance of a strain across media conditions (both Yoch and Yngk have the same TIS). This partially aligns with previous work showing that different SSs yield a range of titers for a given recombinant protein, although the growth characteristics of different strains is not discussed [34, 35]. However, the only variable changing among the Yoch, OG3x, OG6x, UTR1x, UTR3x, and UTR6x strains is the TIS, as the signal and linker sequence (Yoch), plasmid backbone, and recombinant silk sequence are all identical. It is unclear why alterations to the TIS alone would generate substantially different growth characteristics in minimal media (but not LB media) prior to gene induction and such findings are unreported elsewhere in literature. Moreover, the data presented herein does not show a trend relating the TIS monomer sequence or its number of tandem repeats to a strain’s growth characteristics in M9 media. The growth of the Yoch and OG3x strains in M9 both show an extended lag phase (from approximately 3 to 6 h), but the OG6x strain does not. In contrast, the UTR1x and UTR3x strains do not show an extended lag phase in minimal media, but the UTR6x strain does. Indeed, the sequence of the TIS itself may determine how its number of tandem repeats affects a strain’s growth across varying media compositions. Future work should seek to determine if there are differences in plasmid maintenance and basal expression among strains with different SSs and TIS structures, and how these differences change across multiple media compositions.

The ability of the Yngk strain to maintain an A5 4mer titer of 40 mg/L in LB and M9 is significant when compared to the previously tested SoluBL21-pLysS *E. coli* strain, for which an 82% decrease in A5 4mer production was observed when transitioning from LB to M9 [26, 29]. It appears that the Yngk *B. megaterium* strain is more tolerant to minimal media and glucose as a carbon source during recombinant silk production. Our previous work supported the hypothesis that this sensitivity to glucose in the *E. coli* system was due to a combination of a toxic silk product (product toxicity) and a metabolic burden in response to A5 4mer silk production [29]. A metabolic flux analysis showed that the unique primary sequence requirements of the A5 4mer protein (32% glycine, 21% glutamine, 14% proline, and 12% alanine) led to metabolic burdens within the central metabolism and proteogenic pathways of the *E. coli* host, resulting in a reduced citric acid cycle (TCA) flux, acetate overflow, and an upregulated Entner–Doudoroff (ED) pathway [29]. Ultimately, these changes in metabolism led to substantial and harmful reductions in ATP and NADPH synthesis in the *E. coli* host, leading to bottlenecks in amino acid synthesis pathways needed for both biomass and recombinant protein production [29]. Furthermore, it was hypothesized that this metabolic burden was accentuated by the product toxicity of the A5 4mer protein in the cytosol (and thus could potentially be mitigated with secretion) [29]. Supplementing the minimal media with 0.3 g/L of eight amino acids (glutamate, histidine, isoleucine, phenylalanine, proline, tyrosine, lysine, and methionine) was found to relieve these metabolic bottlenecks at least partially in *E. coli* and increase A5 4mer titers by a factor of 2.

**Fig. 5:**
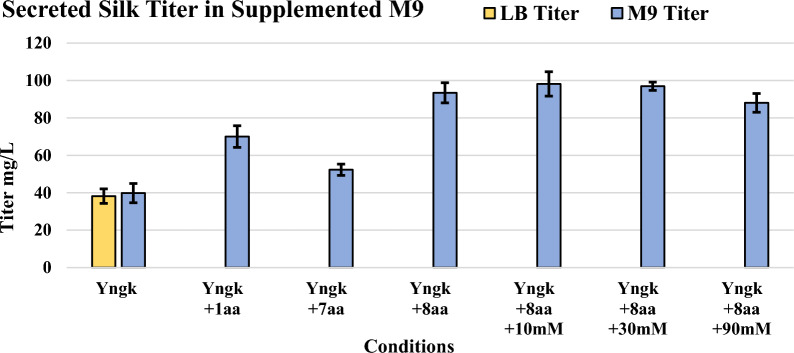
Secreted A5 4mer silk titer for strain Yngk in LB, M9, and supplemented M9 media. Expressions were carried out for 21 h at 37 °C with 1.5% w/v xylose supplemented for recombinant gene induction. All M9 media experiments were performed with 1% w/v glucose as a carbon and energy source. Expressions were performed in supplemented M9 minimal media that contained 0.3 g/L of glutamate (+ 1 aa), 0.3 g/L of histidine, isoleucine, phenylalanine, proline, tyrosine, lysine, and methionine (+ 7 aa), 0.3 g/L of glutamate, histidine, isoleucine, phenylalanine, proline, tyrosine, lysine, and methionine (+ 8 aa), 10 mM sodium acetate at time of induction (+ 10 mM), 30 mM sodium acetate at time of induction (+ 30 mM), and 90 mM sodium acetate at time of induction (+ 90 mM). Error bars represent standard deviations from the mean values of three replicates

Therefore, to investigate the presence of similar metabolic bottlenecks in *B. megaterium* and the potentially positive effect of secretion, expressions were performed with Yngk in M9 media using the same amino acid supplementation protocol that relieved metabolic bottlenecks in *E. coli* [29]*.* When 0.3 g/L of glutamate was applied to the cultures (+ 1aa), the secreted titer increased by 76% from 39.8 ± 5.2 mg/L to 70.0 ± 5.7 mg/L when compared to unaltered M9 media (Fig. 5). Glutamate supplementation was tested in isolation from the other amino acids due to its unique importance in addressing NADPH depletions [29]. Cultures lacking glutamate, but supplemented with 0.3 g/L of histidine, isoleucine, phenylalanine, proline, tyrosine, lysine, and methionine (+ 7aa) yielded a titer of 52.3 ± 3.0 mg/L, a 31% increase compared to unaltered M9. When 0.3 g/L of glutamate and 0.3 g/L of the seven amino acids were supplemented together (+ 8aa) the Yngk strain exhibited at 135% increase in secreted A5 4mer production versus unaltered M9, with a titer of 93.4 ± 5.4 mg/L. This data supports the hypothesis that the ATP and NADPH bottlenecks observed in *E. coli* due during recombinant silk production extend to *B. megaterium*, with NADPH depletion more heavily weighted in *B. megaterium* as shown by its robust response to glutamate supplementation alone (Fig. 5) [29].

However, it appears that there may still be key metabolic differences between the *E. coli* and *B. megaterium* hosts during silk production. In M9 media supplemented with 0.3 g/L of eight amino acids (+ 8aa) the A5 4mer titers for the *E. coli* host are still 57% lower than in LB media [29]. This contrasts with the *B. megaterium* system in which the M9 with eight amino acids outperforms LB media by 145% (Fig. 5). This result, combined with the observation of increased biomass during expression for *B. megaterium* in LB media versus M9 media plus eight amino acids, is also noteworthy when considering the higher titers for the latter (Fig. 4 and 5). It is possible that for the *B. megaterium* system, the amino-acid rich, but carbohydrate depleted, LB media preferentially directs metabolic fluxes to biomass as opposed to silk synthesis through unknown mechanisms. In contrast, in M9 minimal media, the abundance of glucose as a carbon and energy source enables a preferential metabolic flux of supplemented amino acids towards silk synthesis. Indeed, work from Yang et al. demonstrated similar findings to those described here. It was shown that supplementing specific amino acids (< 0.1 g/L of an individual amino acid) in minimal media increased secreted penicillin G amidase production by sevenfold in *B. megaterium*, enabling production levels that surpassed cultivations in complex media [49]. Furthermore, exclusion of the aromatic residues tyrosine, tryptophan, and phenylalanine was found to be critical for increasing secreted production [49]. Thus, a metabolic flux analysis of the novel system developed herein would serve as a worthwhile aspect of future work to characterize these fundamental mechanisms and work towards optimized production.

Notwithstanding, another factor outside of metabolic changes that could explain these unique outcomes in various medias is the repression of the *xylA* promoter by up to 13-fold in *B. megaterium* when glucose is present [48]. The *xylA* promoter controls the production of the T7 promoter for this system, with the T7 promoter directing the expression of the A5 4mer gene. This downregulation of the promoter strength may represent a key strategy for producing disordered recombinant silk proteins in gram-positive species. Indeed, this hypothesis is adjacently supported by our previous work showing that a tight regulation of basal expression promotes favorable A5 4mer titers in *E. coli* [26]*.* Future work should seek to analyze promoters of varying strength and characterize the effects of basal expression within a *B. megaterium* host strain.

Central to the findings for the previously described *E. coli* strain (SoluBL21-pLysS) was the hypothesis that the presence of the toxic A5 4mer product led to an accentuation of metabolic stress, potentially by increasing the strains sensitivity to acetate overflow and oxidative stress [29]. The *E. coli* SoluBL21-pLysS strain is uniquely adapted for disordered, toxic recombinant protein production as it has several mutations within key stress response pathways, including those involved in acetate and oxidative stress [26]. It was found that supplementing the *E. coli* SoluBL21-pLysS cultures with exogenous acetate during A5 4mer expressions in M9 media led to substantial decreases in titer and cell growth [29]. This supported the hypothesis that the A5 4mer exerts a product toxicity, and exogenous acetate may overwhelm the strain’s ability to tolerate the increased stress induced by the presence of a toxic recombinant silk protein in the cytosol [29]. Within this scope is the aspect of intracellular production in the *E. coli* system, whereby the disordered A5 4mer can exert its product toxicity effects on the host through a variety of mechanisms that may include promiscuous binding interactions [26, 55].

Therefore, the secretion of the A5 4mer in the *B. megaterium* system may represent a useful tool to mitigate product toxicity and eliminate its impact on metabolic burdens and titers in a glucose-based media. To investigate this, expression cultures of Yngk in M9 media with 0.3 g/L of eight amino acids (+ 8aa) were supplemented with acetate in an identical fashion to previous experiments performed with *E. coli* SoluBL21-pLysS (10, 30, or 90 mM of exogenous acetate added at time of induction) [29]. Results for strain Yngk show that the previous titer of approximately 93 mg/L in M9 with eight amino acids was maintained across all acetate concentrations tested, with titers during acetate supplementation ranging from 88 to 98 mg/L (Fig. 5). This contrasts with *E. coli* SoluBL21-pLysS in which a 52, 57, and 85% decrease in titer was observed at 10-, 30-, and 90-mM acetate, respectively [29]. Likewise, the final OD600 of strain Yngk at the various acetate levels was approximately equal to that in the baseline media, ranging from 2.4 to 2.7 (Fig. 4). In contrast, the SoluBL21-pLysS strain showed reductions in final OD600 of 28, 28, and 39% at 10-, 30-, and 90-mM acetate, respectively [29]. These results support the hypothesis that secretion can break negative metabolic feedback loops within a microbial host by mitigating the product toxicity of disordered recombinant spidroins. Moreover, secretion of silk proteins is a useful tool when minimal media and a glucose feedstock are desired for a given bioprocess design.

## Conclusions

This work has detailed the creation and study of a novel platform for secretory recombinant silk production using the *Bacillus* genus. Results show that the choice of SS is a primary factor determining success, with the Yngk and Yoch SSs enabling secretory silk production. Interestingly, removal of the SS entirely was found to prevent silk production in the host. Moreover, data indicates that alterations to the translation initiation site in the context of *B. megaterium* and recombinant silk production do not yield the same trends observed for other proteins and gram-positive hosts. Tandem translation initiation sites with varying nucleotide designs did not yield a benefit in titer, however, the presence of multiple start codons did not appear to interfere with proper secretion of the construct through the Sec pathway. This is the first study to report that both the SS and the design of the translation initiation site impacted the ability of recombinant *B. megaterium* to grow in M9 minimal media, through an unknown mechanism. Results support the hypothesis that this *B. megaterium* host suffers from metabolic bottlenecks (ATP and NADPH depletion) in response to silk production that are similar to those characterized in *E. coli,* due to the large glutamine and proline requirements of the A5 4mer. Targeted amino acid supplementation in M9 media appeared to relieve these bottlenecks and enabled a 135–145% increase in titer versus LB or unaltered M9 media. Secretion was found to mitigate the product toxicity of the A5 4mer protein, reduce the host’s sensitivity to acetate stress, and promote favorable outcomes in a glucose-based media. Future work should seek to implement RT-qPCR and metabolic flux analysis to precisely understand changes in this *Bacillus* host system in response to silk production for continued optimization. Likewise, further investigation into a greater range of SSs and promoter strengths is supported by preliminary findings in this work. Additional work should seek to understand the mechanisms behind the effect of tandem translation initiation sites, potentially deciphering the lack of titer increases observed for this host strain and its altered growth characteristics in minimal media when the SS or translation initiation site are modulated. Finally, future work should seek to examine the performance of this system within a bioreactor.

## Materials and methods

### Bacterial strains, media, and culture conditions

All bacterial strains used in this study are listed in Additional file 1: Table S1. *E. coli* and *B. megaterium* were both routinely grown in LB (Lennox broth, Sigma Aldrich, St. Louis, MO). For cloning and plasmid preparation *E. coli* was cultured in shake flasks at 37 °C with shaking at 225 rpm and supplemented with 100 μg/ml ampicillin. In some cases, *B. megaterium* was grown in M9 minimal media supplemented with trace minerals (48 mM M Na_2_HPO_4_, 22 mM KH_2_PO_4_, 18.8 mM NH_4_Cl, 8.6 mM NaCl, 0.1 mM CaCl_2_, 2 mM MgSO_4_, 36 μM FeSO_4_, 4.14 μM MnSO_4_) and 1% w/v glucose as a carbon source. In some cases, *B. megaterium* was grown in the aforementioned M9 media supplemented with 0.3 g/L of glutamate (one amino acid, + 1aa), or 0.3 g/L of histidine, isoleucine, phenylalanine, proline, tyrosine, lysine, and methionine (seven amino acids, + 7aa), or 0.3 g/L of glutamate, histidine, isoleucine, phenylalanine, proline, tyrosine, lysine, and methionine (eight amino acids, + 8aa). In some cases, cultures of *B. megaterium* grown in M9 media with 0.3 g/L of eight amino acids were supplemented with 10, 30, or 90 mM sodium acetate at the time of recombinant gene induction. Cultures of *B. megaterium* containing recombinant gene cassettes were supplemented with tetracycline at 40 μg/ml and chloramphenicol at 25 μg/ml when grown in LB media, or 20 μg/ml tetracycline and 10 μg/ml chloramphenicol when grown in M9 media and M9 media variants.

### Plasmid preparations, transformations, and recombinant silk expressions

A list of all plasmids used in this work and the recombinant silk sequence can be found in Additional file 1: Table S1. To prepare *B. megaterium* strains capable of secreting recombinant silk protein, a variant of the A5 4mer recombinant silk sequence expressed previously in *E. coli* was designed and purchased in the pBluescript II SK( +) vector backbone (Genscript, Piscataway, NJ). The A5 4mer gene was initially cloned into five variants of a pT7 vector backbone using the SalI and SpeI restriction sites. All restriction cloning performed in this work was done with previously described methods [26]. The pT7 vector enables recombinant gene expression using a T7 promoter in *B. megaterium* when the pT7-RNAP vector is simultaneously present in a given strain [35]. The five variants of the pT7 vector used for initial experiments each contain a unique SS and linker sequence prior to the start of the recombinant silk gene to enable secretion of the construct, namely pT7-αamy-A5, pT7-LipA-A5, pT7-Nprm-A5, pT7-Yoch-A5, and pT7-Yngk-A5 (Additional file 1: Table S1) [35]. For creation of strains with multiple tandem translation initiation sites (TISs) the gene fragments of the sequences OG3x, OG6x, UTR1x, UTR3x, UTR6x (Additional file 1: Table S1) were purchased in the pBluescript II SK( +) vector backbone and cloned in the pT7-Yoch-A5 construct using the BspTI and NheI restriction sites. Each new translation initiation sequence contained a copy of the T7 promoter region found on the pT7 vector backbone upstream of the translation initiation sequence to facilitate ease of cloning (T7 promoter sequence not shown in Additional file 1: Table S1). A strain containing the A5 4mer gene but lacking a SS and linker (no secretion strain) was created by purchasing an A5 4mer gene in the pBluescript II SK( +) vector backbone with NheI replacing the original SalI N-terminal restriction site. The construct was then cloned into a pT7-Yoch-A5 backbone using the NheI and SalI sites to remove the Yoch SS and linker and yield pT7-A5. Transformations of vectors into *B. megaterium* were performed according to previous a previously described protoplast protocol [56]. The pT7-RNAP vector was first transformed into *B. megaterium* to form a parent strain (Additional file 1: Table S1, MS941-pRNAP). All pT7 vector variants containing an A5 4mer silk sequence were then transformed into this parent strain for recombinant silk expression and secretion experiments. All colonies of *B. megaterium* used for silk expression were sequenced to confirm proper transformation of the pT7 constructs.

Recombinant silk expressions were carried out by first inoculating 25 ml cultures with a single plated colony and incubating overnight at 37 °C with shaking at 225 rpm. For expression in LB media, overnight cultures were grown in LB media. For expression in M9 media and M9 variants, overnight cultures were grown in either M9 or LB media depending on the specific strain’s ability to grow overnight in M9 media. 300 ml expression cultures (placed in 1-L flasks) were inoculated with 6 ml of an overnight culture and grown at 37 °C with shaking at 225 rpm. Cultures were induced for silk expression via the addition of 0.5% or 1.5% w/v xylose when the OD600 was between 0.275 and 0.325. Cultures were either kept at 37 °C or shifted to 30 °C or 20 °C upon induction and total expression times were 21 h.

### Purification and quantification of recombinant silk

Expression cultures were centrifuged at 7500 rcf for 30 min to separate the culture supernatant from the cell pellet. For analysis on intracellular production, cell pellets were first put through a freeze thaw cycle before being resuspended in 2 ml/per gram cell pellet of B-PER II Bacterial Protein Extraction Reagent (Thermo Fisher Scientific, Waltham, MA) supplemented with 20 mg/ml lysozyme, 1 mg/ml DNaseI, and 1 mM PMSF. The mixture was then incubated at 37 °C with shaking at 225 rpm for two hours before being vortexed for 60 s. The cell lysate was then centrifuged at 7500 rcf for 30 min to pellet the insoluble debris. Both the soluble and insoluble fractions of the lysates were put through nickel chromatography and SDS PAGE to identify if intracellular silk production had occurred using previously described procedures [26]. For analysis on secreted production, culture supernatants were first concentrated from 300 to 25 ml using Amicon centrifugal filters according to the manufacturer’s protocol (Sigma Aldrich, St. Louis, MO). The concentrated supernatants were then put through nickel chromatography and SDS PAGE using previously described methods [26]. Elution fractions after chromatography (for both intracellular and secreted analyses) were sometimes concentrated up to fivefold using Amicon centrifugation filters before SDS PAGE analysis to confirm the absence of recombinant protein in cases where no protein bands were seen on unconcentrated elution fractions. The titers of the secreted and purified A5 4mer silk protein were calculated using the Pierce BCA Protein Assay Kit and the manufacturer’s protocol (Thermo Fisher Scientific, Waltham, MA).

### Supplementary Information

**Additional file 1:** **Table S1**. Strains, plasmids, signal sequences, recombinant silk sequence, and translation initiation sites (sequences). **Figure S1.** SDS PAGE from nickel-chromatography purification on the supernatant of strain UTR6x performed after an expression in LB media. Lanes (1) Protein ladder with kDa values listed to the left (2) Flow through (3) Wash 1 (4) Wash 2 (5) Elution. The purified A5 4mer silk is seen at an apparent molecular weight of approximately 60kDa, which is identical to the secreted A5 4mer produced in the Yoch parent strain. The apparent weight of the secreted silk produced in strain UTR6x is representative of those produced in the OG3x, OG6x, UTR1x, and UTR3x strains.

## Data Availability

No data availability statement available.
