# Supplementary material for: Production and secretion of recombinant spider silk in Bacillus megaterium
Source: Microb Cell Fact. 2024 Jan 26;23:35. doi: 10.1186/s12934-024-02304-5 (PMC10821235; doi:10.1186/s12934-024-02304-5)
Supplement: Supplementary file 1 — Additional file 1: Table S1. Strains, plasmids, signal sequences, recombinant silk sequence, and translation initiation sites (sequences). Figure S1. SDS PAGE from nickel-chromatography purification on the supernatant of strain UTR6x performed after an expression in LB media. Lanes (1) Protein ladder with kDa values listed to the left (2) Flow through (3) Wash 1 (4) Wash 2 (5) Elution. The purified A5 4mer silk is seen at an apparent molecular weight of approximately 60kDa, which is identical to the secreted A5 4mer produced in the Yoch parent strain. The apparent weight of the secreted silk produced in strain UTR6x is representative of those produced in the OG3x, OG6x, UTR1x, and UTR3x strains. [file 12934_2024_2304_MOESM1_ESM.docx]

**Additional Materials**

**Production and Secretion of Recombinant Spider Silk in *Bacillus megaterium***

**Table S1: Strains, plasmids, signal sequences, recombinant silk sequence, and translation initiation sites (sequences).**

| **Strains** | | |
| --- | --- | --- |
| **Strain** | **Description** | **Source** |
| ***E. coli* Strains** |  |  |
| Dh5α | Cloning Strain | Life Technologies |
| ***B. megaterium Strains*** |  |  |
| MS941 | Mutant of DSM319, ∆nprM. | MoBiTec GmbH |
| MS941-pRNAP | Strain MS941 transformed with pT7-RNAP vector. Used as the recipient strain for all pT7 expression vectors. Used for all silk production and secretion experiments. | This work |
| **Plasmids/Other Strains** | | |
| **Plasmid** | **Description** | **Source** |
| pBluescript II SK(+) | Cloning vector for storage and propagation of A5 4mer recombinant silk sequence and translation initiation site variations in *E coli* Dh5α | Genscript |
| pT7 | Shuttle vector for cloning in *E. coli* and recombinant gene expression in *B. megaterium* using a T7 promoter | MoBiTec GmbH |
| pT7-RNAP | Shuttle vector for cloning in *E. coli* and  xylose-inducible expression of the T7 promoter *B.*  *megaterium* | MoBiTec GmbH |
| pT7-αamy-A5  (αamy) | Derivative of pT7 with an α-amy signal sequence and linker inserted prior to the A5 4mer silk sequence. | This work |
| pT7-LipA-A5  (LipA) | Derivative of pT7 with a LipA signal sequence and linker inserted prior to the A5 4mer silk sequence. | This work |
| pT7-NprM-A5  (NprM) | Derivative of pT7 with a NprM signal sequence and linker inserted prior to the A5 4mer silk sequence. | This work |
| pT7-Yoch-A5  (Yoch) | Derivative of pT7 with a Yoch signal sequence and linker inserted prior to the A5 4mer silk sequence. | This work |
| pT7-Yngk-A5  (Yngk) | Derivative of pT7 with an Yngk signal sequence and linker inserted prior to the A5 4mer silk sequence. | This work |
| pT7-A5  (No secretion) | Derivative of pT7-Yoch-A5 with the Yoch signal sequence and linker removed. | This work |
| pT7-OG3x-A5  (OG3x) | Derivative of pT7-Yoch-A5 with 3 tandem copies of the original translation initiation site (including start codon), followed by a Yoch signal sequence and linker inserted prior to the A5 4mer silk sequence. | This work |
| pT7-OG6x-A5  (OG6x) | Derivative of pT7-Yoch-A5 with 6 tandem copies of the original translation initiation site (including start codon), followed by a Yoch signal sequence and linker inserted prior to the A5 4mer silk sequence. | This work |
| pT7-UTR1x-A5  (UTR1x) | Derivative of pT7-Yoch-A5 with a new translation initiation site (UTR), followed by a Yoch signal sequence and linker inserted prior to the A5 4mer silk sequence. | This work |
| pT7-UTR3x-A5  (UTR3x) | Derivative of pT7-UTR1x-A5 with 3 tandem copies of the UTR translation initiation site (including start codon), followed by a Yoch signal sequence and linker inserted prior to the A5 4mer silk sequence. | This work |
| pT7-UTR6x-A5  (UTR6x) | Derivative of pT7-UTR1x-A5 with 6 tandem copies of the UTR translation initiation site (including start codon), followed by a Yoch signal sequence and linker inserted prior to the A5 4mer silk sequence. | This work |
| **Signal Sequences** | | |
| **Name** | **Translated Sequence** | **Source** |
| α-amy | Signal: MKWKRTSMLLILLLLFGSSASA  Linker: QDHKD | [35] |
| LipA | Signal: MKKVLMAFIICLSLILSVLAAPPSGAKA  Linker: ESVHN | [35] |
| NprM | Signal: MKKKKQALKVLLSVGILSSSFAFAHTSSA  Linker: APNNV | [35] |
| Yoch | Signal: MKKTMITFSLVLMSLFGVASGASA  Linker: ATNTY | [35] |
| Yngk | Signal: MYIKKCIGSILFLLLFCSSALPAKA  Linker: DVPTT | [35] |
| **Recombinant Silk Sequence** | | |
| **Name** | **Translated Sequence** | **Source** |
| A5 4mer (version produced in *B. megaterium)*  n = 4 | (GPGQQAAAAAGPGQQGPGQQGPGQQGPGEQGPGSG)**n**  HHHHHH | This work |
| A5 4mer (version produced in *E. coli)*  n = 4 | MGHHHHHHHHHHSSGHIDDDDKHMLEHMPG  (GPGQQAAAAAGPGQQGPGQQGPGQQGPGEQGPGSG)**n**  TSGS | [26] |
| **Translation Initiation Sequences*** | | |
| **Name** | **Nucleotide Sequence** | **Source** |
| OG3x | TAATTAACCAAGGAGGTGAAAGGTAGC**ATG**  AAATTAACCAAGGAGGTGAAAGGTAGC**ATG**  AAATTAACCAAGGAGGTGAAAGGTAGC**ATG**  GCTAGC | This work |
| OG6x | TAATTAACCAAGGAGGTGAAAGGTAGC**ATG**  AAATTAACCAAGGAGGTGAAAGGTAGC**ATG**  AAATTAACCAAGGAGGTGAAAGGTAGC**ATG**  AAATTAACCAAGGAGGTGAAAGGTAGC**ATG**  AAATTAACCAAGGAGGTGAAAGGTAGC**ATG**  AAATTAACCAAGGAGGTGAAAGGTAGC**ATG**  GCTAGC | This work |
| UTR1x | TGTATATTAGAAAGGAGGAATATATAA**ATG**  GCTAGC | This work |
| UTR3x | TGTATATTAGAAAGGAGGAATATATAAA**ATG**  GTATATTGGAAAGGAGGAATATATAAA**ATG**  GTATATTGGAAAGGAGGAATATATAAA**ATG**  GCTAGC | This work |
| UTR6x | TGTATATTAGAAAGGAGGAATATATAAA**ATG**  GTATATTGGAAAGGAGGAATATATAAA**ATG**  GTATATTGGAAAGGAGGAATATATAAA**ATG**  GTATATTGGAAAGGAGGAATATATAAA**ATG**  GTATATTGGAAAGGAGGAATATATAAA**ATG**  GTATATTGGAAAGGAGGAATATATAAA**ATG**  GCTAGC | This work |

*Nucleotide sequence shows base pairs directly after T7 promoter region until directly before the first codon of the Yoch signal sequence (Yoch signal sequence and linker sequence used for all translation initiation sequence variations). Some sequences contain multiple start codons (bolded).

**Figure S1: SDS PAGE from nickel-chromatography purification on the supernatant of strain UTR6x performed after an expression in LB media. Lanes (1) Protein ladder with kDa values listed to the left (2) Flow through (3) Wash 1 (4) Wash 2 (5) Elution. The purified A5 4mer silk is seen at an apparent molecular weight of approximately 60kDa, which is identical to the secreted A5 4mer produced in the Yoch parent strain. The apparent weight of the secreted silk produced in strain UTR6x is representative of those produced in the OG3x, OG6x, UTR1x, and UTR3x strains.**


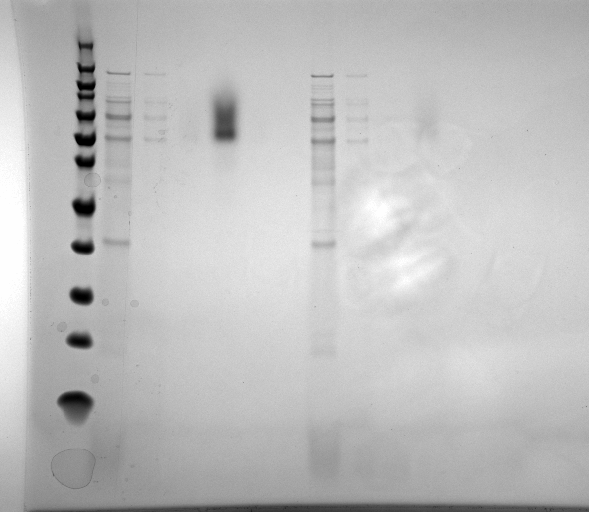


10

15

20

30

40

50

60

80

2600

1600

1100

3.5

**1 2 3 4 5**

**References (same numbering as in main text)**

26. Connor, A.; Wigham, C.; Bai, Y.; Rai, M.; Nassif, S.; Koffas, M.; Zha, R. H. Novel Insights into Construct Toxicity, Strain Optimization, and Primary Sequence Design for Producing Recombinant Silk Fibroin and Elastin-like Peptide in E. Coli. *Metab. Eng. Commun.* **2023**, *16*, e00219.  [DOI:10.1016/j.mec.2023.e00219](https://doi.org/10.1016/j.mec.2023.e00219).

35. Kalbarczyk, K. Z.; Mazeau, E. J.; Rapp, K. M.; Marchand, N.; Koffas, M. A. G.; Collins, C. H. Engineering Bacillus Megaterium Strains to Secrete Cellulases for Synergistic Cellulose Degradation in a Microbial Community. *ACS Synth. Biol.* **2018**, *7* (10), 2413–2422. [DOI:10.1021/acssynbio.8b00186](https://doi.org/10.1021/acssynbio.8b00186).
